# Marker effect p-values for single-step GWAS with the algorithm for proven and young in large genotyped populations

**DOI:** 10.1186/s12711-024-00925-3

**Published:** 2024-08-22

**Authors:** Natália Galoro Leite, Matias Bermann, Shogo Tsuruta, Ignacy Misztal, Daniela Lourenco

**Affiliations:** 1Department of Animal and Dairy Science, University of Georgia, Athens, GA 30602 USA

## Abstract

**Background:**

Single-nucleotide polymorphism (SNP) effects can be backsolved from ssGBLUP genomic estimated breeding values (GEBV) and used for genome-wide association studies (ssGWAS). However, obtaining p-values for those SNP effects relies on the inversion of dense matrices, which poses computational limitations in large genotyped populations. In this study, we present a method to approximate SNP p-values for ssGWAS with many genotyped animals. This method relies on the combination of a sparse approximation of the inverse of the genomic relationship matrix (${\mathbf{G}}_{\mathbf{A}\mathbf{P}\mathbf{Y}}^\mathbf{-1}$) built with the algorithm for proven and young ($\text{APY}$) and an approximation of the prediction error variance of SNP effects which does not require the inversion of the left-hand side (LHS) of the mixed model equations. To test the proposed p-value computing method, we used a reduced genotyped population of 50K genotyped animals and compared the approximated SNP p-values with benchmark p-values obtained with the direct inverse of LHS built with an exact genomic relationship matrix (${\mathbf{G}}^\mathbf{-1})$. Then, we applied the proposed approximation method to obtain SNP p-values for a larger genotyped population composed of 450K genotyped animals.

**Results:**

The same genomic regions on chromosomes 7 and 20 were identified across all p-value computing methods when using 50K genotyped animals. In terms of computational requirements, obtaining p-values with the proposed approximation reduced the wall-clock time by 38 times and the memory requirement by ten times compared to using the exact inversion of the LHS. When the approximation was applied to a population of 450K genotyped animals, two new significant regions on chromosomes 6 and 14 were uncovered, indicating an increase in GWAS detection power when including more genotypes in the analyses. The process of obtaining p-values with the approximation and 450K genotyped individuals took 24.5 wall-clock hours and 87.66GB of memory, which is expected to increase linearly with the addition of noncore genotyped individuals.

**Conclusions:**

With the proposed method, obtaining p-values for SNP effects in ssGWAS is computationally feasible in large genotyped populations. The computational cost of obtaining p-values in ssGWAS may no longer be a limitation in extensive populations with many genotyped animals.

**Supplementary Information:**

The online version contains supplementary material available at 10.1186/s12711-024-00925-3.

## Background

The single-step genomic best linear unbiased prediction (ssGBLUP) has been successfully implemented in the routine genetic evaluation of several livestock species [1–3]. The vast adoption of ssGBLUP is associated with the straightforward and simultaneous evaluation of populations composed of genotyped and non-genotyped animals, the non-requirement of pseudo phenotypes, the decrease in biases attributed to double counting and genomic preselection, and reliable estimation of breeding values for complex genetic models [4].

Although ssGBLUP is a breeding value-based method that provides genomic estimated breeding values (GEBVs), obtaining single-nucleotide polymorphism (SNP) effects from this method may also be valuable when investigating how genome segments are associated with important traits. When that is the case, SNP effects can be easily obtained from a linear transformation of GEBVs following formulas presented by VanRaden [5], Strandén and Garrick [6], and Wang et al. [7]. Besides SNP effects, the proportion of genetic variance explained by single SNPs or by SNP windows can help identify important regions of the genome in single-step genome-wide association studies (ssGWAS) [8–10]. However, this procedure does not consider the uncertainty of the SNP effect estimation, making it more difficult to replicate findings from ssGWAS [9, 10]. To overcome this problem, Aguilar et al. [11] presented formulas for obtaining frequentist p-values for ssGWAS as an extension of the ideas previously presented by Gualdrón Duarte et al. [12], Bernal Rubio et al. [13], and Lu et al. [14] in the ssGBLUP context. The authors also showed that p-values could be successfully obtained within a reasonable computational time for a large Angus population accounting for roughly 1M phenotyped individuals, 1500 genotyped sires, and about 1.8M animals in the pedigree.

The formulas presented by Aguilar et al. [11] require obtaining the prediction error variance of the SNP effects ($\text{var}\left({\widehat{\text{a}}}_{\text{i}}\right)$) which relies on obtaining the breeding value prediction error (co)variance for genotyped animals (${\mathbf{C}}^{{\mathbf{u}}_\mathbf{2}{\mathbf{u}}_\mathbf{2}}$) through the inversion of the left-hand side (LHS) of the mixed model equations. The inversion of such a matrix becomes challenging as the number of traits and genotyped animals increases. With genomic information, the LHS is associated with a very dense block represented by the inverse of the genomic relationship matrix (${\mathbf{G}}^\mathbf{-1}$), which is hard to obtain directly for more than 100K genotyped animals [4]. One approach to deal with the computation limits with large genotyped populations is to use a sparse approximation of ${\mathbf{G}}^\mathbf{-1}$ created by the algorithm for proven and young (APY) [15]. In APY, the genotyped individuals are split into two sets. The set of genotyped animals representing all genomic variation is called “core” (non-redundant information); the remaining animals are “noncore” (redundant information). Then, the GEBVs of noncore animals are conditioned on the GEBVs of core animals, making ${\mathbf{G}}^\mathbf{-1}$ very sparse. Apart from increasing the sparseness of ${\mathbf{G}}^\mathbf{-1}$, Bermann et al. [16] showed that, with APY, obtaining $\text{var}\left({\widehat{\text{a}}}_{\text{i}}\right)$ can be reduced to components only associated with the prediction error covariance of GEBVs for the core set (${\mathbf{C}}^{{{\mathbf{u}}_{\mathbf{2}_{\mathbf{C}}}}{\mathbf{u}}_{\mathbf{2}_{\mathbf{C}}}}$), drastically reducing the dimensionality of matrices involved in calculations to obtain $\text{var}\left({\widehat{\text{a}}}_{\text{i}}\right)$. However, in the formulas shown by Bermann et al. [16], obtaining ${\mathbf{C}}^{{{\mathbf{u}}_{\mathbf{2}_{\mathbf{C}}}}{\mathbf{u}}_{\mathbf{2}_{\mathbf{C}}}}$ still requires a direct inversion of the LHS with all genotyped animals. Even though ${\mathbf{G}}^\mathbf{-1}$ is sparser with APY, components in the LHS of single-step equations such as the inverse of the pedigree relationship matrix for genotyped animals (${\mathbf{A}}_{22}^\mathbf{-1}$) are still dense, thus implying that computation limits for the inversion of LHS might still exist.

One way to overcome this problem is to obtain an approximated prediction error (co)variance of GEBVs for the APY core set $({\mathbf{C}}^{{{\mathbf{u}}_{\mathbf{2}_{\mathbf{C}}}}{\mathbf{u}}_{{\mathbf{2}_{\mathbf{C}}}_{{\varvec{a}}{\varvec{p}}{\varvec{p}}{\varvec{r}}{\varvec{o}}{\varvec{x}}}}})$ that does not require the inversion of the LHS. For that, an extension of the algorithm proposed by Misztal and Wiggans [17] that accounts for genomic information with APY was presented by Bermann et al. [18]. In this algorithm, Bermann et al. [18] showed that ${\mathbf{C}}^{{{\mathbf{u}}_{\mathbf{2}_{\mathbf{C}}}}{\mathbf{u}}_{{\mathbf{2}_{\mathbf{C}}}_{{\varvec{a}}{\varvec{p}}{\varvec{p}}{\varvec{r}}{\varvec{o}}{\varvec{x}}}}}$ can be obtained with a block-sparse inversion of ${\mathbf{G}}^\mathbf{-1}$ with APY (${\mathbf{G}}_{\mathbf{A}\mathbf{P}\mathbf{Y}}^\mathbf{-1}$) plus a diagonal matrix of contributions from phenotypes and pedigree relationships. Empirical results shown by the authors demonstrate that ${\mathbf{C}}^{{{\mathbf{u}}_{\mathbf{2}_{\mathbf{C}}}}{\mathbf{u}}_{{\mathbf{2}_{\mathbf{C}}}_{{\varvec{a}}{\varvec{p}}{\varvec{p}}{\varvec{r}}{\varvec{o}}{\varvec{x}}}}}$ is obtained in a few minutes for an Angus population with about 300K genotyped animals. Moreover, they showed that, although computation complexity increases cubically with the number of core animals, that remains linear for the noncore set. Thus, combining APY and ${\mathbf{C}}^{{{\mathbf{u}}_{\mathbf{2}_{\mathbf{C}}}}{\mathbf{u}}_{{\mathbf{2}_{\mathbf{C}}}_{{\varvec{a}}{\varvec{p}}{\varvec{p}}{\varvec{r}}{\varvec{o}}{\varvec{x}}}}}$ should enable the approximation of p-values for ssGWAS for large genotyped populations within a feasible amount of time and computational resources. Therefore, this study presents a method to approximate SNP p-values for large genotyped populations based on APY. The performance of the proposed method was tested against the regular way to compute p-values using the exact inverse of LHS with ${\mathbf{G}}^\mathbf{-1}$ or ${\mathbf{G}}_{\mathbf{A}\mathbf{P}\mathbf{Y}}^\mathbf{-1}$ with 50K genotyped animals. Then, the final test involved applying the proposed approximation method to a dataset with 450K genotyped animals.

## Methods

### Theory

In ssGBLUP, SNP effects can be obtained from backsolving GEBV using a linear transformation [5–7]:

1 $$\widehat{\mathbf{a}}=(1-\upbeta )\text{ b}\frac{{\upsigma }_{\text{u}}^\mathbf{2}}{{\upsigma }_{\text{a}}^\mathbf{2}}\mathbf{Z}\mathbf{^{\prime}}{\mathbf{G}}^\mathbf{-1}\widehat{\mathbf{u}},$$

where $\widehat{\mathbf{a}}$ is the vector of SNP effects, $\upbeta$ is the blending parameter (5%) to avoid singularity problems in $\mathbf{G}$ [5]; $\text{b}$ is a tuning parameter [19], ${\upsigma }_{\text{u}}^\mathbf{2}$ is the genetic variance, ${\upsigma }_{\text{a}}^\mathbf{2}$ is SNP variance, $\mathbf{Z}$ is a matrix of SNP content centered by two times the allele frequency (p), $\widehat{\mathbf{u}}$ is the vector of GEBVs, and ${\mathbf{G}}^\mathbf{-1}$ is the inverse of the genomic relationship matrix, with $\mathbf{G}$ constructed as the type I of VanRaden [5]:

2 $$\mathbf{G}=\left(1-\upbeta \right)\left(\mathbf{11}^{\mathbf{^{\prime}}}{\alpha }+\text{b}\frac{\mathbf{Z}{\mathbf{Z}}^{\mathbf{^{\prime}}}}{2\sum {\text{p}}_{\text{i }}\left(1-{\text{p}}_{\text{i}}\right)}\right)+\upbeta {\mathbf{A}}_\mathbf{22},$$

where α and $\text{b}$ are tuning parameters to assure the compatibility between $\mathbf{G}$ and ${\mathbf{A}}_{22}$ [19], and other elements were defined above.

Once SNP effects ($\widehat{\mathbf{a}})$ are estimated, the p-value for the ith SNP can be obtained as shown by Aguilar et al. [11]:

3 $${\text{p}-\text{value}}_{\text{i}}=2\left(1-\upphi \left(\left|\frac{{\widehat{\text{a}}}_{\text{i}}}{\text{sd}({\widehat{\text{a}}}_{\text{i}})}\right|\right)\right),$$

where $\text{sd}({\widehat{\text{a}}}_{\text{i}})$ is the square root of the variance of the ith SNP effect estimate obtained as [12]:

4 $$\text{var}\left({\widehat{\text{a}}}_{\text{i}}\right)= \frac{1}{2\sum {\text{p}}_{\text{i }}\left(1-{\text{p}}_{\text{i}}\right)}\left(1-\upbeta \right)\text{b}{\mathbf{z}}_{\mathbf{i}}^{\mathbf{{\prime}}}{\mathbf{G}}^\mathbf{-1}\left(\mathbf{G}{\upsigma }_{\text{u}}^{2}-{\mathbf{C}}^{{\mathbf{u}}_\mathbf{2}{\mathbf{u}}_\mathbf{2}}\right){\mathbf{G}}^\mathbf{-1}{\mathbf{z}}_{\mathbf{i}}\left(1-\upbeta \right)\text{b}\frac{1}{2\sum {\text{p}}_{\text{i }}\left(1-{\text{p}}_{\text{i}}\right)},$$

with ${\mathbf{C}}^{{\mathbf{u}}_\mathbf{2}{\mathbf{u}}_\mathbf{2}}$ referring to the matrix of GEBV prediction error (co)variance for genotyped animals, and other parameters are defined above. The computation of $\text{var}\left({\widehat{\text{a}}}_{\text{i}}\right)$ is restrained by the costs associated with obtaining ${\mathbf{G}}^\mathbf{-1}$ and ${\mathbf{C}}^{{\mathbf{u}}_\mathbf{2}{\mathbf{u}}_\mathbf{2}}$. Those components result from the inversion of dense matrices of high dimension, and obtaining them becomes unfeasible with large genotyped populations [11, 20].

The computational limitations of obtaining ${\mathbf{G}}^\mathbf{-1}$ can be overcome by replacing this matrix with a sparse approximation built with APY [15]. With APY, a small set of genotyped animals (core) is chosen, and the relationship of the remaining animals (noncore) is obtained by recursions on the core set with linear computing cost. The inverse of the genomic relationship matrix with APY is constructed as follows:

$${\mathbf{G}}_{\mathbf{A}\mathbf{P}\mathbf{Y}}^\mathbf{-1}=\left[\begin{array}{cc}{\mathbf{G}}_{\mathbf{c}\mathbf{c}}^\mathbf{-1}& \mathbf{0}\\ \mathbf{0}& \mathbf{0}\end{array}\right]+\left[\begin{array}{c}-{\mathbf{G}}_{\mathbf{c}\mathbf{c}}^\mathbf{-1}{\mathbf{G}}_{\mathbf{c}\mathbf{n}}\\ \mathbf{I}\end{array}\right]{\mathbf{M}}_{\mathbf{n}\mathbf{n}}^\mathbf{-1}\left[\begin{array}{cc}-{\mathbf{G}}_{\mathbf{n}\mathbf{c}}{\mathbf{G}}_{\mathbf{c}\mathbf{c}}^\mathbf{-1}& \mathbf{I}\end{array}\right]$$

5 $$=\left[\begin{array}{cc}{\mathbf{G}}_{\mathbf{c}\mathbf{c}}^\mathbf{-1}+{\mathbf{G}}_{\mathbf{c}\mathbf{c}}^\mathbf{-1}{\mathbf{G}}_{\mathbf{c}\mathbf{n}}{\mathbf{M}}_{\mathbf{n}\mathbf{n}}^\mathbf{-1}{\mathbf{G}}_{\mathbf{n}\mathbf{c}}{\mathbf{G}}_{\mathbf{c}\mathbf{c}}^\mathbf{-1}& -{\mathbf{G}}_{\mathbf{c}\mathbf{c}}^\mathbf{-1}{\mathbf{G}}_{\mathbf{c}\mathbf{n}}{\mathbf{M}}_{\mathbf{n}\mathbf{n}}^\mathbf{-1}\\ -{\mathbf{M}}_{\mathbf{n}\mathbf{n}}^\mathbf{-1}{\mathbf{G}}_{\mathbf{n}\mathbf{c}}{\mathbf{G}}_{\mathbf{c}\mathbf{c}}^\mathbf{-1}& {\mathbf{M}}_{\mathbf{n}\mathbf{n}}^\mathbf{-1}\end{array}\right]=\left[\begin{array}{cc}{\mathbf{G}}^{\mathbf{c}\mathbf{c}}& {\mathbf{G}}^{\mathbf{c}\mathbf{n}}\\ {\mathbf{G}}^{\mathbf{n}\mathbf{c}}& {\mathbf{M}}_{\mathbf{n}\mathbf{n}}^\mathbf{-1}\end{array}\right],$$

  where ${\mathbf{G}}_{\mathbf{c}\mathbf{c}}^\mathbf{-1}$ and ${\mathbf{M}}_{\mathbf{n}\mathbf{n}}^\mathbf{-1}$ are the inverses of the full genomic relationship matrix for core and diagonal for noncore animals, respectively, and ${\mathbf{G}}_{\mathbf{c}\mathbf{n}}$ is the genomic relationship matrix between core and noncore animals. The elements of the matrix ${\mathbf{M}}_{\mathbf{n}\mathbf{n}}^\mathbf{-1}$ are obtained as:

6 $${\mathbf{m}}_{\text{nn},\text{j}}=\text{diag}\left\{{\mathbf{g}}_{\text{jj}}- {\mathbf{g}}_{\text{jc}}^{{{\prime}}}{\mathbf{G}}_{\mathbf{c}\mathbf{c}}^\mathbf{-1}{\mathbf{g}}_{\text{cj}}\right\},$$

where ${\mathbf{g}}_{\mathbf{j}\mathbf{j}}$ is the diagonal element of ${\mathbf{G}}_{\mathbf{n}\mathbf{n}}$ for the jth animal, and ${\mathbf{g}}_{\text{jc}}$ is the relationship between the jth noncore animal with core animals. With APY, the need to invert a dense and high dimensional $\mathbf{G}$ is reduced to only inverting the genomic relationship matrix for core animals $({\mathbf{G}}_{\mathbf{c}\mathbf{c}}$) [Eq. (5)], which for most livestock species or breeds contains less than 15K animals [21, 22].

Beyond the reductions in computing costs of obtaining ${\mathbf{G}}^\mathbf{-1}$, Bermann et al. [16] showed that, with APY, estimating the $\text{var}\left({\widehat{\text{a}}}_{\text{i}}\right)\text{ as in Eq }(4)$ is reduced to components only associated with the core animals:

7 $$\text{var}\left({\widehat{\text{a}}}_{\text{i}}\right)= \frac{1}{2\sum {\text{p}}_{\text{i }}\left(1-{\text{p}}_{\text{i}}\right)}\left(1-{\alpha }\right)\text{b}{\mathbf{z}}_{\mathbf{c}\mathbf{j}}^{\mathbf{{\prime}}}{\mathbf{G}}_{\mathbf{C}\mathbf{C}}^\mathbf{-1}\left({\mathbf{G}}_{\mathbf{C}\mathbf{C}}{\upsigma }_{\text{u}}^\mathbf{2}-{\mathbf{C}}^{{{\mathbf{u}}_{\mathbf{2}_{\mathbf{C}}}}{\mathbf{u}}_{\mathbf{2}_{\mathbf{C}}}}\right){\mathbf{G}}_{\mathbf{C}\mathbf{C}}^\mathbf{-1}{\mathbf{z}}_{\mathbf{c}\mathbf{j}}\left(1-{\alpha }\right)\text{b}\frac{1}{2\sum {\text{p}}_{\text{i }}\left(1-{\text{p}}_{\text{i}}\right)},$$

where ${\mathbf{z}}_{\mathbf{c}\mathbf{j}}$ is the jth column of the $\mathbf{Z}$ matrix for core animals, and ${\mathbf{C}}^{{{\mathbf{u}}_{\mathbf{2}_{\mathbf{C}}}}{\mathbf{u}}_{\mathbf{2}_{\mathbf{C}}}}$ is the prediction error (co)variance matrix of GEBVs for core animals, and other elements are as defined before. However, obtaining ${\mathbf{C}}^{{{\mathbf{u}}_{\mathbf{2}_{\mathbf{C}}}}{\mathbf{u}}_{\mathbf{2}_{\mathbf{C}}}}$, still depends on the inversion of a high dimension LHS, which might yet limit computations as model complexity and the number of genotyped animals increase. To overcome this limitation, an approximation of the prediction error (co)variance matrix of GEBVs for core animals (${\mathbf{C}}^{{{\mathbf{u}}_{\mathbf{2}_{\mathbf{C}}}}{\mathbf{u}}_{{\mathbf{2}_{\mathbf{C}}}_{{\varvec{a}}{\varvec{p}}{\varvec{p}}{\varvec{r}}{\varvec{o}}{\varvec{x}}}}})$ can be obtained as follows [18]:

8 $${\mathbf{C}}^{{{\mathbf{u}}_{\mathbf{2}_{\mathbf{C}}}}{\mathbf{u}}_{{\mathbf{2}_{\mathbf{C}}}_{{\varvec{a}}{\varvec{p}}{\varvec{p}}{\varvec{r}}{\varvec{o}}{\varvec{x}}}}} = {\left({\mathbf{D}}_{\mathbf{c}}+\uplambda {\mathbf{G}}^{\mathbf{c}\mathbf{c}}-\uplambda {\mathbf{G}}^{\mathbf{c}\mathbf{n}}{\left(\uplambda {\mathbf{M}}_{\mathbf{n}\mathbf{n}}^\mathbf{-1}+{\mathbf{D}}_{\mathbf{n}}\right)}^\mathbf{-1}\uplambda {\mathbf{G}}^{\mathbf{n}\mathbf{c}}\right)}^\mathbf{-1},$$

where $\uplambda =\frac{{\upsigma }_{\text{e}}^\mathbf{2}}{{\upsigma }_{\text{u}}^\mathbf{2}}$, ${\upsigma }_{\text{e}}^\mathbf{2}$ is the residual variance, and ${\mathbf{D}}_{\mathbf{c}}$ and ${\mathbf{D}}_{\mathbf{n}}$ are the blocks for core and noncore animals from the diagonal matrix $\mathbf{D}$ constructed as [17, 23]:

9 $$\mathbf{D}\approx {\mathbf{W}}{{^{\prime}}}{\left(\mathbf{I}-\mathbf{X}({\mathbf{X}}{{^{\prime}}}\mathbf{X}\right)}^\mathbf{-1}\mathbf{X}{^{\prime}})\mathbf{W},$$

where $\mathbf{W}$ and $\mathbf{X}$ are incidence matrices for animal and fixed effects. Therefore, when combining APY and ${\mathbf{C}}^{{{\mathbf{u}}_\mathbf{2}}_{\mathbf{C}}{\mathbf{u}}_{{\mathbf{2}_{\mathbf{C}}}_{{\varvec{a}}{\varvec{p}}{\varvec{p}}{\varvec{r}}{\varvec{o}}{\varvec{x}}}}}$ the $\text{var}\left({\widehat{\text{a}}}_{\text{i}}\right)$ can be approximated as:

10 $$\text{var}\left({\widehat{\text{a}}}_{\text{i}}\right)\approx \frac{1}{2\sum {\text{p}}_{\text{i }}\left(1-{\text{p}}_{\text{i}}\right)}\left(1-{\alpha }\right)\text{b}{\mathbf{z}}_{\mathbf{c}\mathbf{j}}^{\mathbf{^{\prime}}}{\mathbf{G}}_{\mathbf{C}\mathbf{C}}^\mathbf{-1}\left({\mathbf{G}}_{\mathbf{C}\mathbf{C}}{\upsigma }_{\text{u}}^\mathbf{2}-{\mathbf{C}}^{{{\mathbf{u}}_{\mathbf{2_{\mathbf{C}}}}}{\mathbf{u}}_{{\mathbf{2}_{\mathbf{C}}}_{{\varvec{a}}{\varvec{p}}{\varvec{p}}{\varvec{r}}{\varvec{o}}{\varvec{x}}}}}\right){\mathbf{G}}_{\mathbf{C}\mathbf{C}}^\mathbf{-1}{\mathbf{z}}_{\mathbf{c}\mathbf{j}}\left(1-{\alpha }\right)\text{b}\frac{1}{2\sum {\text{p}}_{\text{i }}\left(1-{\text{p}}_{\text{i}}\right)},$$

where all parameters were defined above. Equation (10) implies that when APY and ${\mathbf{C}}^{{{\mathbf{u}}_{\mathbf{2}_{\mathbf{C}}}}{\mathbf{u}}_{{\mathbf{2}_{\mathbf{C}}}_{{\varvec{a}}{\varvec{p}}{\varvec{p}}{\varvec{r}}{\varvec{o}}{\varvec{x}}}}}$ are combined, SNP p-values can be calculated with matrices only associated with core animals and without the requirement of inverting the LHS, thus potentially lifting the current computational limitations for large genotyped populations.

Therefore, the proposed method to approximate p-values for SNP involves the following steps:

1. Save ${\mathbf{G}}_{\text{APY}}^\mathbf{-1}$ and components of ${\mathbf{A}}_{22}^\mathbf{-1}$ [24] in disk (PREGSF90 from BLUPF90 software suite; Misztal et al. [25]);
2. Obtain GEBVs based on APY by reading the saved matrices (BLUP90IOD3 from BLUPF90 software suite);
3. Compute ${\mathbf{C}}^{{{\mathbf{u}}_{\mathbf{2}_{\mathbf{C}}}}{\mathbf{u}}_{{\mathbf{2}_{\mathbf{C}}}_{{\varvec{a}}{\varvec{p}}{\varvec{p}}{\varvec{r}}{\varvec{o}}{\varvec{x}}}}}$ using block sparse inversion as in Bermann et al. (2022b) (ACCF90GS2 from BLUPF90 software suite);
4. Use ${\mathbf{C}}^{{{\mathbf{u}}_{\mathbf{2}_{\mathbf{C}}}}{\mathbf{u}}_{{\mathbf{2}_{\mathbf{C}}}_{{\varvec{a}}{\varvec{p}}{\varvec{p}}{\varvec{r}}{\varvec{o}}{\varvec{x}}}}}$ and ${\mathbf{G}}_{\text{cc}}^\mathbf{-1}$ from ${\mathbf{G}}_{\text{APY}}^\mathbf{-1}$ to compute $\text{var}\left({\widehat{\text{a}}}_{\text{i}}\right)$ as in Eq. (10) (POSTGSF90 from BLUPF90 software suite);
5. Backsolve SNP effects from GEBVs obtained in step 2 as in Eq. (1) with ${\mathbf{G}}_{\text{APY}}^\mathbf{-1}$; compute SNP p-values_i_ as in Eq. (3) by using the square root of $\text{var}\left({\widehat{\text{a}}}_{\text{i}}\right)$ obtained in step 4 (POSTGSF90 from BLUPF90 software suite).

### Dataset

The American Angus Association (Saint Joseph, MO) provided the dataset to test the proposed method to approximate p-values for SNP. A total of 844,726 animals born from 2012 to 2017 were scored for post-weaning gain (PWG). Phenotyped animals were produced by 93,161 sires and 812,292 cows and were distributed into 64,889 contemporary groups. Genomic information on 39,744 SNP (after quality control) was available for 450,673 animals born from 2012 to 2018. Of the genotyped animals, 217,434 were also phenotyped, whereas the remaining animals only contributed with genotypes and pedigree. Pedigree information was available for all phenotyped and genotyped animals up to 3 generations of relationships, summing up 1,837,789 records.

#### Reduced dataset

In this study we aimed to compare different p-value computing methods, where p-values obtained from a direct inversion of the LHS were used as benchmark (see Statistical analyses for more details). Due to the computational limitations of inverting the LHS, a reduced genomic subset of 50K randomly selected genotyped animals was created. As the subset of 50K genotyped animals were selected randomly, the sampling process was repeated three times, thus three reduced genotype subsets were created. Phenotypic information was kept complete for all replicates. However, the number of animals in the pedigree varied slightly (from 1,576,112 to 1,576,738). This small variation is due to the creation of the pedigree in a way that it traces back three generations for phenotyped and genotyped animals in the dataset, and for the reduced datasets, genotyped animals varied because of sampling.

### Statistical model

A single-trait animal model was used for the estimation of PWG GEBVs as follows:

11 $$\mathbf{y} =\mathbf{X}{\varvec{\upbeta}}+\mathbf{W}\mathbf{u} +\mathbf{e},$$

where $\mathbf{y}$ is the vector of PWG phenotypes; ${\varvec{\upbeta}}$ is the vector containing the fixed effect of contemporary groups; $\mathbf{u}$ is the vector of random additive genetic effects; $\mathbf{e}$ is the vector of random residuals; and $\mathbf{X}$, and $\mathbf{W}$ are incidence matrices for the effects contained in ${\varvec{\upbeta}}$ and $\mathbf{u}$, respectively. Random effects were distributed as ${\text{e}} \sim {\mkern 1mu} N{\text{(}}{\mathbf{0}}{\text{, }}{\mathbf{I}}\sigma _{{\text{e}}}^{{\mathbf{2}}} {\text{) and u}} \sim N{\text{(}}{\mathbf{0}}{\text{, }}{\mathbf{H}}\sigma _{{\text{u}}}^{{\mathbf{2}}} {\text{)}}$, where $\mathbf{I}$ is an identity matrix, and $\mathbf{H}$ is the realized relationship matrix for genotyped and non-genotyped animals in ssGBLUP, with inverse constructed as shown by Aguilar et al. [26]:

12 $${\mathbf{H}}^\mathbf{-1}={\mathbf{A}}^\mathbf{-1}+\left[\begin{array}{cc}\mathbf{0}& \mathbf{0}\\ \mathbf{0}& {\mathbf{T}}^\mathbf{-1}-{\mathbf{A}}_{22}^\mathbf{-1}\end{array}\right],$$

where ${\mathbf{A}}^\mathbf{-1}$ is the inverse of the pedigree relationship matrix and ${\mathbf{T}}^\mathbf{-1}$ is equal to ${\mathbf{G}}_{\mathbf{A}\mathbf{P}\mathbf{Y}}^\mathbf{-1}$[Eq. (5)] for genetic analyses with APY, and equal to ${\mathbf{G}}^\mathbf{-1}$ [Eq. (2)] otherwise. The ${\mathbf{A}}_{22}^\mathbf{-1}$ was built as defined before.

### Statistical analyses

#### Comparison between p-value computing methods in a small genotyped population

In this set of analyses, we aimed to compare exact p-values obtained with a regular ${\mathbf{G}}^\mathbf{-1}$ and ${\mathbf{C}}^{{\mathbf{u}}_\mathbf{2}{\mathbf{u}}_\mathbf{2}}$ (Exact_ $\text{Ginv}$) as a benchmark [Eqs. (3) and (4)], with p-values with ${\mathbf{G}}_{\mathbf{A}\mathbf{P}\mathbf{Y}}^\mathbf{-1}$ and exact ${\mathbf{C}}^{{{\mathbf{u}}_{\mathbf{2}_{\mathbf{C}}}}{\mathbf{u}}_{\mathbf{2}_{\mathbf{C}}}}$ (Exact_GinvAPY) [Eqs. (3) and (7)], and p-values obtained with ${\mathbf{G}}_{\mathbf{A}\mathbf{P}\mathbf{Y}}^\mathbf{-1}$ and ${\mathbf{C}}^{{{\mathbf{u}}_{\mathbf{2}_{\mathbf{C}}}}{\mathbf{u}}_{{\mathbf{2}_{\mathbf{C}}}_{{\varvec{a}}{\varvec{p}}{\varvec{p}}{\varvec{r}}{\varvec{o}}{\varvec{x}}}}}$ (Approx_GinvAPY) [Eqs. (3) and (10)]. Because the p-values from Exact_ $\text{Ginv}$ and Exact_GinvAPY require obtaining the inverses of the genomic relationship matrix and of the LHS, a reduced subset of 50K was used to ensure computation feasibility and fair comparisons.

For methods involving APY (i.e., Exact_GinvAPY and Approx_GinvAPY), the APY core was composed of 13,030 genotypes, which corresponded to the number of eigenvalues explaining 98% of the genetic variance in $\mathbf{G}$. This was obtained applying the singular value decomposition of $\mathbf{Z}$ composed of all genotypes available (i.e., 450 K) [22]. The selection of core animals was made at random. This decision was supported by previous studies showing the performance of random core selection in comparison to alternative selection strategies [3, 27, 28]. Moreover, as the genotyped set was reduced to 50K for this set of analyses, that also reduced opportunities to high contrasts for the core-noncore compositions (i.e., core composed of sires with high accuracies or with large number of genotyped progeny).

After all analyses performed, p-value computing methods were compared based on the Pearson correlation of computed p-values, SNP effects, $\text{var}\left({\widehat{\text{a}}}_{\text{i}}\right)$, in addition to the inspection of Manhattan and QQ plot results. Wall-clock time and Resident Set Size (RSS) memory requirements were also recorded.

#### Application of Approx_GinvAPY in a large genotyped population

In the second set of analyses, we aimed to calculate p-values with ${\mathbf{G}}_{\mathbf{A}\mathbf{P}\mathbf{Y}}^\mathbf{-1}$ and ${\mathbf{C}}^{{{\mathbf{u}}_{\mathbf{2}_{\mathbf{C}}}}{\mathbf{u}}_{{\mathbf{2}_{\mathbf{C}}}_{{\varvec{a}}{\varvec{p}}{\varvec{p}}{\varvec{r}}{\varvec{o}}{\varvec{x}}}}}$ with the full set of 450K genotyped animals (Approx_GinvAPY450K). Note that, Approx_GinvAPY and Approx_GinvAPY450K comprised the same p-value computing method, the only difference is the size of the genotype set (50K vs. 450K, respectively). For straightforward interpretations, within the same replicate, the core sets in Approx_GinvAPY450K were composed of the same set of animals as with the analyses with the reduced dataset. For example, within each replicate, the core set in Exact_ $\text{Ginv}$, Approx_GinvAPY, and Approx_GinvAPY450K consisted of the same genotyped animals.

To evaluate the robustness of the proposed p-value computing method regarding core composition, we ran an extra scenario where the APY core of Approx_GinvAPY450K was composed of genotyped animals with the highest estimated breeding value (EBV) accuracies in the population (Approx_GinvAPY450K_high-acc). EBV accuracies were obtained in a previous step without including genomic information, which means their merit was mainly based on progeny contributions. The core dimension was kept constant at 13,030 genotypes.

Elapsed wall-clock time and RSS memory requirements were recorded. The inspection of Manhattan and QQ plots results were used to evaluate the soundness of the approximation applied to the full genotype set. A summary of the information available for all analyses with reduced or full genotype sets is displayed in Table 1.

**Table 1:** Number of records per source of information for all p-value computing methods

Method^a^	Genotypes	Core	Pedigree	Phenotypes
Exact_Ginv	50,000	13,030	1,576,738^b^	844,726
Exact_GinvAPY	50,000	13,030	1,576,738^b^	844,726
Approx_GinvAPY	50,000	13,030	1,576,738^b^	844,726
Approx_GinvAPY450K	450,673	13,030	1,837,789	844,726

^a^SNP p-values obtained from a data set of 50K genotyped with ${\mathbf{G}}^\mathbf{-1}$ and ${\mathbf{C}}^{{\mathbf{u}}_\mathbf{2}{\mathbf{u}}_\mathbf{2}}$ (Exact_Ginv), ${\mathbf{G}}_{\mathbf{A}\mathbf{P}\mathbf{Y}}^\mathbf{-1}$ and ${\mathbf{C}}^{{{\mathbf{u}}_{\mathbf{2}_{\mathbf{C}}}}{\mathbf{u}}_{\mathbf{2}_{\mathbf{C}}}}$(Exact_GinvAPY), and ${\mathbf{G}}_{\mathbf{A}\mathbf{P}\mathbf{Y}}^\mathbf{-1}$ and ${\mathbf{C}}^{{{\mathbf{u}}_{\mathbf{2}_{\mathbf{C}}}}{\mathbf{u}}_{{\mathbf{2}_{\mathbf{C}}}_{{\varvec{a}}{\varvec{p}}{\varvec{p}}{\varvec{r}}{\varvec{o}}{\varvec{x}}}}}$(Approx_GinvAPY). Approx_GinvAPY450K refers to the Approx_GinvAPY method applied to a genotyped population of 450K animals

^b^Because pedigree traced back three generations of relationships from phenotyped and genotyped animals, the number of animals in the pedigree slightly varied from 1,576,112 to 1,576,738 between replicates

For all GWAS analyses performed in this study, a significance level of 5% adjusted by multiple testing via Bonferroni correction was used as a SNP rejection threshold, i.e., -log(0.05/m); where m (39,744) is the number of markers in the SNP panel. As the SNP panel density was kept constant throughout this study, this implies a fixed rejection threshold for all sets of analyses and comparisons. Moreover, all analyses were performed with software from the BLUPF90 software suite [25] on an Intel(R) Xeon(R) CPU E5-2650 v4 @ 2.20 GHz server with 24 threads. New implementations for obtaining p-values with ${\mathbf{G}}_{\mathbf{A}\mathbf{P}\mathbf{Y}}^\mathbf{-1}$ and ${\mathbf{C}}^{{{\mathbf{u}}_{\mathbf{2}_{\mathbf{C}}}}{\mathbf{u}}_{{\mathbf{2}_{\mathbf{C}}}_{{\varvec{a}}{\varvec{p}}{\varvec{p}}{\varvec{r}}{\varvec{o}}{\varvec{x}}}}}$ were available in modified versions of BLUP90IOD3 and ACCF90GS2 [29].

## Results and discussion

Using ssGWAS for association studies in farm animal populations increases the detection power because it considers phenotypic information from non-genotyped individuals, allows for complex models involving multiple traits and environmental and genetic correlated effects, and does not rely on pseudo phenotypes [10, 30, 31]. However, computing SNP p-values from ssGWAS still depends on the inversion of the LHS and should become prohibited with increasing data dimensionality. In this study, we approach the challenge of computing p-value in populations with an increasing number of genotyped individuals with APY. For that, we compared three methods to calculate p-values, which consisted of obtaining p-values with (1) a regular ${\mathbf{G}}^\mathbf{-1}$ and ${\mathbf{C}}^{{\mathbf{u}}_\mathbf{2}{\mathbf{u}}_\mathbf{2}}$ (Exact_ $\text{Ginv}$), (2) with ${\mathbf{G}}_{\mathbf{A}\mathbf{P}\mathbf{Y}}^\mathbf{-1}$ and exact ${\mathbf{C}}^{{{\mathbf{u}}_{\mathbf{2}_{\mathbf{C}}}}{\mathbf{u}}_{\mathbf{2}_{\mathbf{C}}}}$** (**Exact_GinvAPY**)**, and (3) an efficient method combining ${\mathbf{G}}_{\mathbf{A}\mathbf{P}\mathbf{Y}}^\mathbf{-1}$ and ${\mathbf{C}}^{{{\mathbf{u}}_{\mathbf{2}_{\mathbf{C}}}}{\mathbf{u}}_{{\mathbf{2}_{\mathbf{C}}}_{{\varvec{a}}{\varvec{p}}{\varvec{p}}{\varvec{r}}{\varvec{o}}{\varvec{x}}}}}$ (Approx_GinvAPY). We later evaluated the performance of the Approx_GinvAPY method when applied to a large genotyped population comprised of around 450K individuals with a random core composition (Approx_GinvAPY450K) and with a core composed of genotyped animals with the highest EBV accuracies in the population (Approx_GinvAPY450K_high-acc).

### Comparison between p-value computing methods in a small genotyped population

**Fig. 1:**
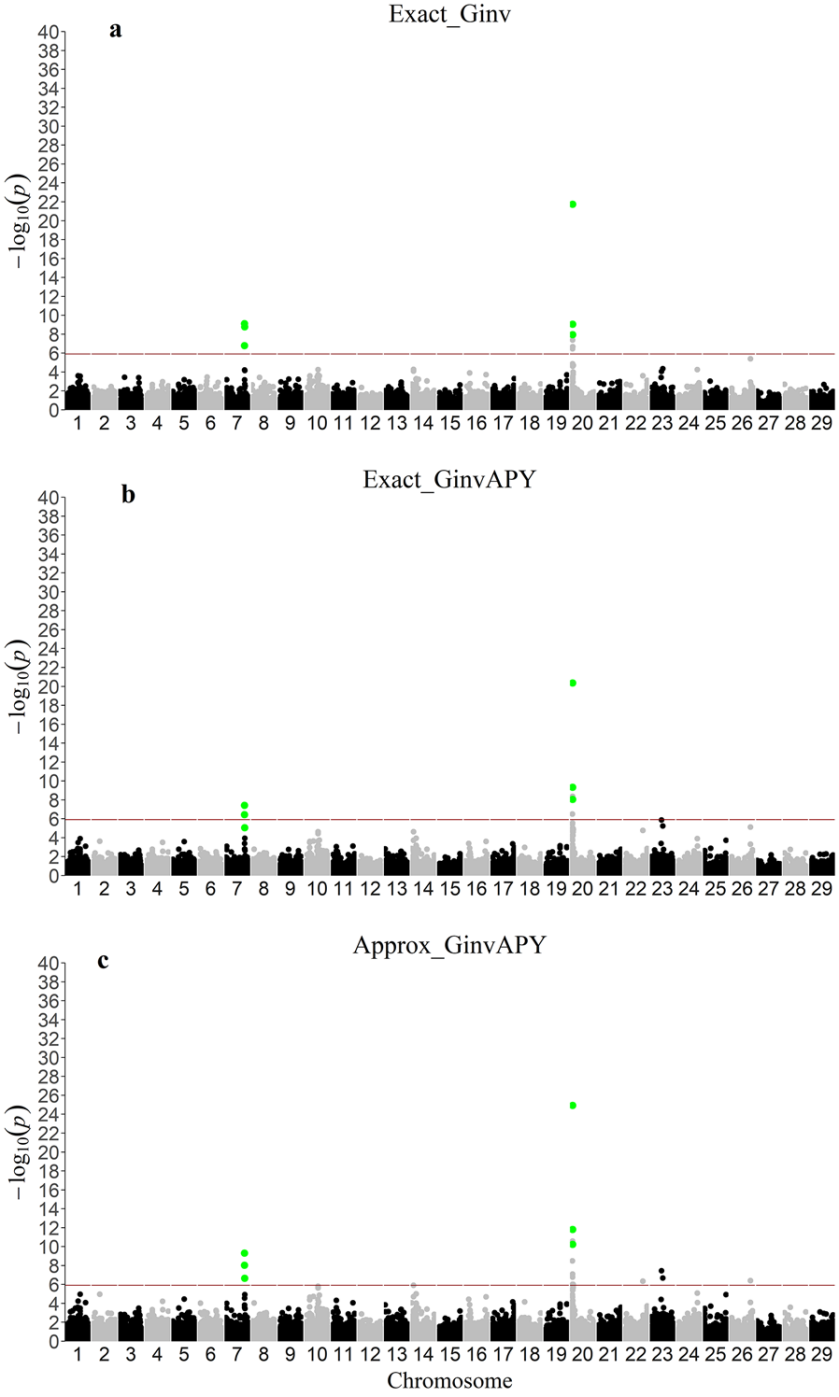
Manhattan plots for all p-value computing methods with a reduced data set in replicate 1. Single-step genome-wide association study for post-weaning weight with p-values obtained from a data set of 50K genotyped animals with (**A**) ${\mathbf{G}}^\mathbf{-1}$ and ${\mathbf{C}}^{{\mathbf{u}}_\mathbf{2}{\mathbf{u}}_\mathbf{2}}$ (Exact_Ginv), (**B**) ${\mathbf{G}}_{\mathbf{A}\mathbf{P}\mathbf{Y}}^\mathbf{-1}$ and ${\mathbf{C}}^{{{\mathbf{u}}_{\mathbf{2}_{\mathbf{C}}}}{\mathbf{u}}_{\mathbf{2}_{\mathbf{C}}}}$(Exact_GinvAPY), and (**C**) ${\mathbf{G}}_{\mathbf{A}\mathbf{P}\mathbf{Y}}^\mathbf{-1}$ and ${\mathbf{C}}^{{{\mathbf{u}}_{\mathbf{2}_{\mathbf{C}}}}{\mathbf{u}}_{{\mathbf{2}_{\mathbf{C}}}_{{\varvec{a}}{\varvec{p}}{\varvec{p}}{\varvec{r}}{\varvec{o}}{\varvec{x}}}}}$(Approx_GinvAPY) in replicate 1; SNPs highlighted in green represent the three most significant SNP in the two peaks found with Exact_Ginv (benchmark)

**Fig. 2:**
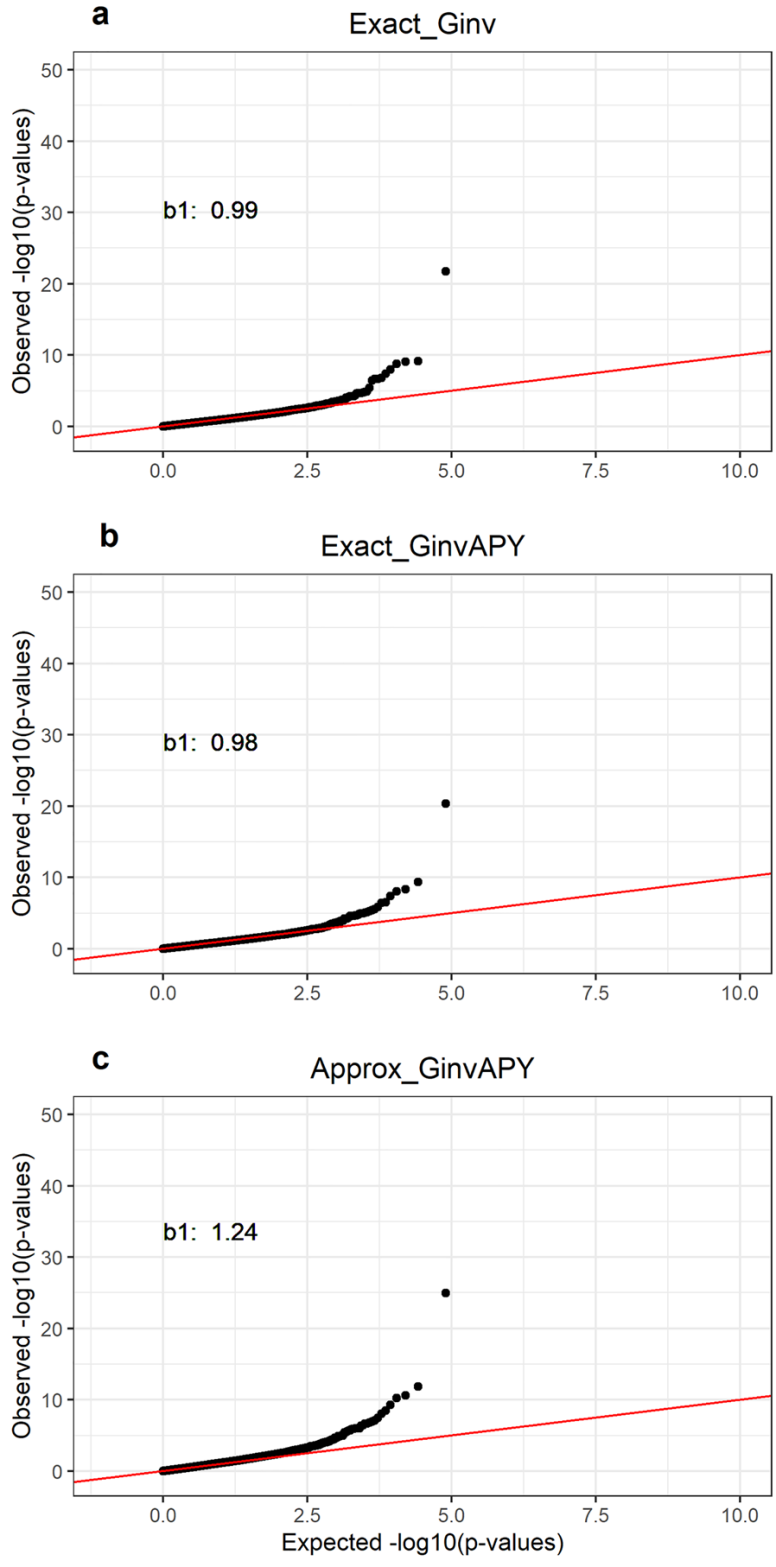
QQ plots for all p-value computing methods with a reduced data set in replicate 1. QQ plots for p-values obtained from a data set of 50K genotyped animals with **A** ${\mathbf{G}}^\mathbf{-1}$ and ${\mathbf{C}}^{{\mathbf{u}}_\mathbf{2}{\mathbf{u}}_\mathbf{2}}$ (Exact_Ginv), **B** ${\mathbf{G}}_{\mathbf{A}\mathbf{P}\mathbf{Y}}^\mathbf{-1}$ and ${\mathbf{C}}^{{{\mathbf{u}}_{\mathbf{2}_{\mathbf{C}}}}{\mathbf{u}}_{\mathbf{2}_{\mathbf{C}}}}$(Exact_GinvAPY), and **C** ${\mathbf{G}}_{\mathbf{A}\mathbf{P}\mathbf{Y}}^\mathbf{-1}$ and ${\mathbf{C}}^{{{\mathbf{u}}_{\mathbf{2}_{\mathbf{C}}}}{\mathbf{u}}_{{\mathbf{2}_{\mathbf{C}}}_{{\varvec{a}}{\varvec{p}}{\varvec{p}}{\varvec{r}}{\varvec{o}}{\varvec{x}}}}}$(Approx_GinvAPY) in replicate 1

Manhattan and QQ plots for all investigated p-value computing methods in replicate 1 are shown in Figs. 1 and 2, respectively. Manhattan and QQ plots for replicates 2 and 3 are provided in Additional file 1: Figures S1, S2, S3, and S4. Across all methods and replicates, two significant peaks were identified on chromosomes 7 and 20 for PWG. For the peak on chromosome 7, the same top three SNPs were identified across all methods. However, for the peak on chromosome 20 only the first top SNP was consistent across p-value computing methods; the second and third top SNP slightly varied across neighboring SNPs within a 2Mb window (Fig. 1, Additional file 1: Figures S1 and S2).

Despite the overall correct identification of the same SNPs with all p-values computing methods, the Approx_GinvAPY method resulted in a higher deviation of p-values from the null hypothesis in comparison to results from Exact_Ginv (Fig. 2, Additional file 1: Figures S3 and S4). Across replicates, the slope of the QQ plot for Exact_Ginv and Exact_GinvAPY was nearly constant at 0.99 (0.99 ± 0.01 and 0.99 ± 0.02, respectively), while the slope of the QQ plot for Approx_GinvAPY increased to 1.25 ± 0.03, indicating an overestimation of -log10(p-values) with our proposed method. In practice, this overestimation can be corrected by the genomic control method proposed by Devlin and Roeder [32]

Correlations of p-values, SNP effects, $\text{var}\left({\widehat{\text{a}}}_{\text{i}}\right)$ across p-value computing methods are displayed in Table 2. Between APY-based methods and Exact_Ginv, correlations were, on average across replicates, constant at 0.82 for p-values, 0.88 for SNP effects, and ranged from 0.91 to 0.92 for $\text{var}\left({\widehat{\text{a}}}_{\text{i}}\right)$. When only significant p-values were considered, the correlation was increased to 0.91 (Table 2). In contrast, between APY-based methods, the correlation for all p-values, significant p-values, SNP effects, and $\text{var}\left({\widehat{\text{a}}}_{\text{i}}\right)$, and p-values approached unity (from 0.99 to 1.00) (Table 2). Those results demonstrate the goodness of the approximation of ${\mathbf{C}}^{{{\mathbf{u}}_{\mathbf{2}_{\mathbf{C}}}}{\mathbf{u}}_{\mathbf{2}_{\mathbf{C}}}}$ (${\mathbf{C}}^{{{\mathbf{u}}_{\mathbf{2}_{\mathbf{C}}}}{\mathbf{u}}_{{\mathbf{2}_{\mathbf{C}}}_{{\varvec{a}}{\varvec{p}}{\varvec{p}}{\varvec{r}}{\varvec{o}}{\varvec{x}}}}}$) (i.e., Exact_GinvAPY vs. Approx_GinvAPY), but also indicate an increase in noise mostly sourced from the use of APY (Exact_Ginv vs. Exact_GinvAPY and Approx_GinvAPY). When approximations are used, errors can be accumulated, especially when multiple steps are involved. For example, for obtaining SNP effects with ssGBLUP, GEBVs are backsolved into SNP effects [Eq. (1)]. For APY-based methods, GEBVs have small changes compared to using ${\mathbf{G}}^\mathbf{-1}$ [33]. Then, the GEBVs are backsolved with a formula that also involves ${\mathbf{G}}_{\mathbf{A}\mathbf{P}\mathbf{Y}}^\mathbf{-1}$. Therefore, potential errors can be accumulated, especially for Approx_GinvAPY, where approximation algorithms are involved. A result from this increase in noise with approximated methods can be demonstrated in Figs. 1, S1, and S2, where few SNPs on chromosomes 22, 23, 26, and 29 achieved significance level without a clear linkage disequilibrium trail with Approx_GinvAPY [34].

**Table 2:** Person correlation between all (above diagonal) and significant^a^ (below diagonal) p-values, SNP effects, and variance of estimated SNP effects across methods

Method^b^	p-value		
	Exact_Ginv	Exact_GinvAPY	Approx_GinvAPY
Exact_Ginv		0.82 ± 0.02	0.82 ± 0.02
Exact_GinvAPY	0.91 ± 0.02		1.00 ± 0.00
Approx_GinvAPY	0.91 ± 0.02	1.00 ± 0.00	

$\text{var}\left({\widehat{\text{a}}}_{\text{i}}\right)$	p-value		
	Exact_Ginv	Exact_GinvAPY	Approx_GinvAPY
	Exact_Ginv	Exact_GinvAPY	Approx_GinvAPY
Exact_Ginv		0.91 ± 0.00	0.92 ± 0.00
Exact_GinvAPY			0.99 ± 0.00
Approx_GinvAPY			

${\widehat{\text{a}}}_{\text{i}}$	p-value		
	Exact_Ginv	Exact_GinvAPY	Approx_GinvAPY
	Exact_Ginv	Exact_GinvAPY	Approx_GinvAPY
Exact_Ginv		0.88 ± 0.01	0.88 ± 0.01
Exact_GinvAPY			1.00 ± 0.00
Approx_GinvAPY			

^a^Significant SNPs were defined based on Exact_Ginv across replicates. ^b^Methods refer to SNP p-values obtained from a data set of 50K genotyped animals with ${\mathbf{G}}^\mathbf{-1}$ and ${\mathbf{C}}^{{\mathbf{u}}_\mathbf{2}{\mathbf{u}}_\mathbf{2}}$ (Exact_Ginv), ${\mathbf{G}}_{\mathbf{A}\mathbf{P}\mathbf{Y}}^\mathbf{-1}$ and ${\mathbf{C}}^{{{\mathbf{u}}_{\mathbf{2}_{\mathbf{C}}}}{\mathbf{u}}_{\mathbf{2}_{\mathbf{C}}}}$(Exact_GinvAPY), and ${\mathbf{G}}_{\mathbf{A}\mathbf{P}\mathbf{Y}}^\mathbf{-1}$ and ${\mathbf{C}}^{{{\mathbf{u}}_{\mathbf{2}_{\mathbf{C}}}}{\mathbf{u}}_{{\mathbf{2}_{\mathbf{C}}}_{{\varvec{a}}{\varvec{p}}{\varvec{p}}{\varvec{r}}{\varvec{o}}{\varvec{x}}}}}$(Approx_GinvAPY)

When obtaining p-values with Approx_GinvAPY estimation noise can be associated with two uncertainty measurements, the first being APY. The algorithm for proven and young is based on the theory that genomic information is limited, and that all genetic variation is contained in a set of independent chromosome segments within a population. Given that a core group of animals would contain those segments, the GEBVs of noncore animals in the population could be estimated from the GEBVs of core animals in addition to an error term ${{\varvec{\Phi}}}_{\mathbf{n}}$ (${\mathbf{u}}_{\text{n}}={\mathbf{G}}_{\mathbf{n}\mathbf{c}}{\mathbf{G}}_{\mathbf{c}\mathbf{c}}^\mathbf{-1}{\mathbf{u}}_{\text{c}}+ {{\varvec{\Phi}}}_{\mathbf{n}}$), which is expected to approach zero when the core size approaches the rank of $\mathbf{G}$ [16, 35]. Note that, when p-values are backsolved with Eq. (1), ${\mathbf{G}}^\mathbf{-1}$ is replaced by ${\mathbf{G}}_{\mathbf{A}\mathbf{P}\mathbf{Y}}^\mathbf{-1}$ and $\widehat{\mathbf{u}}$ is the vector of GEBVs obtained with ${\mathbf{G}}_{\mathbf{A}\mathbf{P}\mathbf{Y}}^\mathbf{-1}$ composing the LHS. Therefore, those two components are affected by the approximation with APY. The second measurement of uncertainty comes from computing ${\mathbf{C}}^{{{\mathbf{u}}_{\mathbf{2}_{\mathbf{C}}}}{\mathbf{u}}_{{\mathbf{2}_{\mathbf{C}}}_{{\varvec{a}}{\varvec{p}}{\varvec{p}}{\varvec{r}}{\varvec{o}}{\varvec{x}}}}}$. As shown by Misztal and Wiggans [17], the off-diagonal elements are not considered during the absorption of environmental effects into the mixed model equations for constructing $\mathbf{D}$. Thus, is expected that, with Approx_GinvAPY, there is a slight increase in noise, especially when the core set and data are small.

The elapsed wall-clock time and RSS memory requirement across p-value computing methods are shown in Table 3. Despite ssGWAS results being similar among methods, computing times varied considerably. The average total elapsed wall-clock was 106.76h for Exact_Ginv, 110.98h for Exact_GinvAPY, and was reduced to 2.83h with Approx_GinvAPY (Table 3). Therefore, compared to Exact_Ginv, the run time with Approx_GinvAPY was reduced by approximately 38 times. The RSS memory requirement also varied across p-value computing methods; its peak was 159.66GB, 178.30GB, and 16.62GB for Exact_Ginv, Exact_GinvAPY, and Approx_GinvAPY, respectively. Compared to Exact_Ginv, the peak RSS memory requirement for obtaining p-values with Approx_GinvAPY was about tenfold smaller.

**Table 3:** Elapsed wall-clock time and Resident Set Size (RSS) memory requirement for all p-values computation methods

Method^a^	Software	Elapsed time, h:min	SD	Peak of memory, GB	SD
	PREGSF90	1:01	0:27	107.28	0.00
Exact_Ginv	BLUPF90IOD3	93.13	21:20	159.66	0.88
	POSTGSF90	12:31	0:21	145.39	0.58
	Total/Max	106:46		159.66	
	PREGSF90	0:20	0:01	9.07	0.00
Exact_GinvAPY	BLUPF90IOD3	108:45	22:33	178.30	0.00
	POSTGSF90	1:53	0:39	44.83	0.00
	Total/Max	110:59		178.30	
	PREGSF90	0:39	0:16	9.07	0.00
	BLUPF90IOD3	0:54	0:21	4.53	0.00
Approx_GinvAPY	ACCF90GS2	0:04	0:02	9.07	0.00
	POSTGSF90	1:50	0:06	16.62	0.00
	Total/Max	2:50		16.62	

^a^SNP p-values obtained from a data set of 50K genotyped with ${\mathbf{G}}^\mathbf{-1}$ and ${\mathbf{C}}^{{\mathbf{u}}_\mathbf{2}{\mathbf{u}}_\mathbf{2}}$ (Exact_Ginv), ${\mathbf{G}}_{\mathbf{A}\mathbf{P}\mathbf{Y}}^\mathbf{-1}$ and ${\mathbf{C}}^{{{\mathbf{u}}_{\mathbf{2}_{\mathbf{C}}}}{\mathbf{u}}_{\mathbf{2}_{\mathbf{C}}}}$(Exact_GinvAPY), and ${\mathbf{G}}_{\mathbf{A}\mathbf{P}\mathbf{Y}}^\mathbf{-1}$ and ${\mathbf{C}}^{{{\mathbf{u}}_{\mathbf{2}_{\mathbf{C}}}}{\mathbf{u}}_{{\mathbf{2}_{\mathbf{C}}}_{{\varvec{a}}{\varvec{p}}{\varvec{p}}{\varvec{r}}{\varvec{o}}{\varvec{x}}}}}$(Approx_GinvAPY)

^b^Resident Set Size (RSS) memory. Values are displayed as average and standard deviations among three replicates

The most computationally demanding scenario was Exact_GinvAPY, with the computation of p-values taking a total wall-clock time run of 110.98h and a peak of RSS memory requirement of 178.30GB. Even though APY increases the sparsity of ${\mathbf{G}}^\mathbf{-1}$ by ignoring the relationships between noncore animals, it still requires the storage of intermediate matrices and vectors. Moreover, the computational advantage with APY comes mainly from the block implementation with the preconditioned conjugate gradient (PCG) method, as shown by Masuda et al. [36]. However, in Exact_GinvAPY, the LHS is still explicitly inverted, which does not use the sparse properties of ${\mathbf{G}}_{\mathbf{A}\mathbf{P}\mathbf{Y}}^\mathbf{-1}$ [37]. Although Exact_GinvAPY has a similar computing performance as Exact_Ginv, results from this method are helpful in this study to illustrate the feasibility of accurately computing p-values with ${\mathbf{G}}_{\mathbf{A}\mathbf{P}\mathbf{Y}}^\mathbf{-1}$.

### Application of Approx_GinvAPY in a large genotyped population

**Fig. 3:**
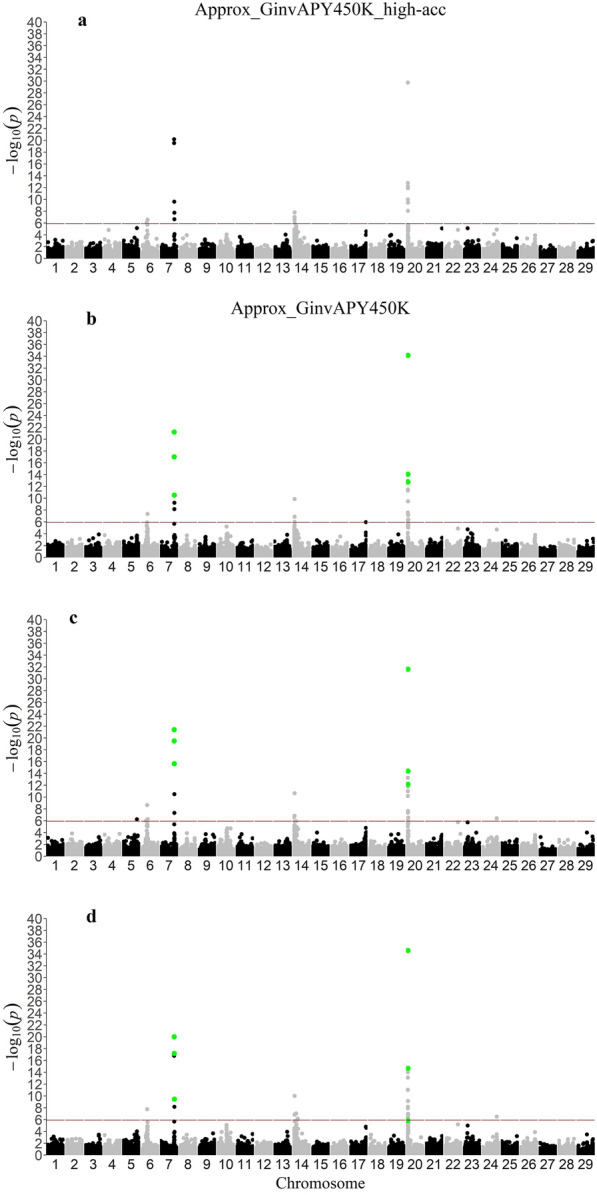
Single-step genome-wide association study for post-weaning weight using Approx_GinvAPY450K and Approx_GinvAPY450K_high-acc. Single-step genome-wide association study for post-weaning weight using Approx_GinvAPY450K in **A** replicate 1, **B** replicate 2, **C** replicate 3, and **D** using Approx_GinvAPY450K_high-acc; SNP highlighted in green represent the three most significant SNP in the two peaks found in with Exact_Ginv with a reduced genotype dataset. Approx_GinvAPY450K refers to the method where SNP p-values are obtained from a data set of 450K genotyped animals with ${\mathbf{G}}_{\mathbf{A}\mathbf{P}\mathbf{Y}}^\mathbf{-1}$ and ${\mathbf{C}}^{{{\mathbf{u}}_{\mathbf{2}_{\mathbf{C}}}}{\mathbf{u}}_{{\mathbf{2}_{\mathbf{C}}}_{{\varvec{a}}{\varvec{p}}{\varvec{p}}{\varvec{r}}{\varvec{o}}{\varvec{x}}}}}$ and where the APY core set if chosen at random; Approx_GinvAPY450K_high-acc refers to the Approx_GinvAPY450K when the APY core is composed of animals with the highest EBV accuracy in the population

**Fig. 4:**
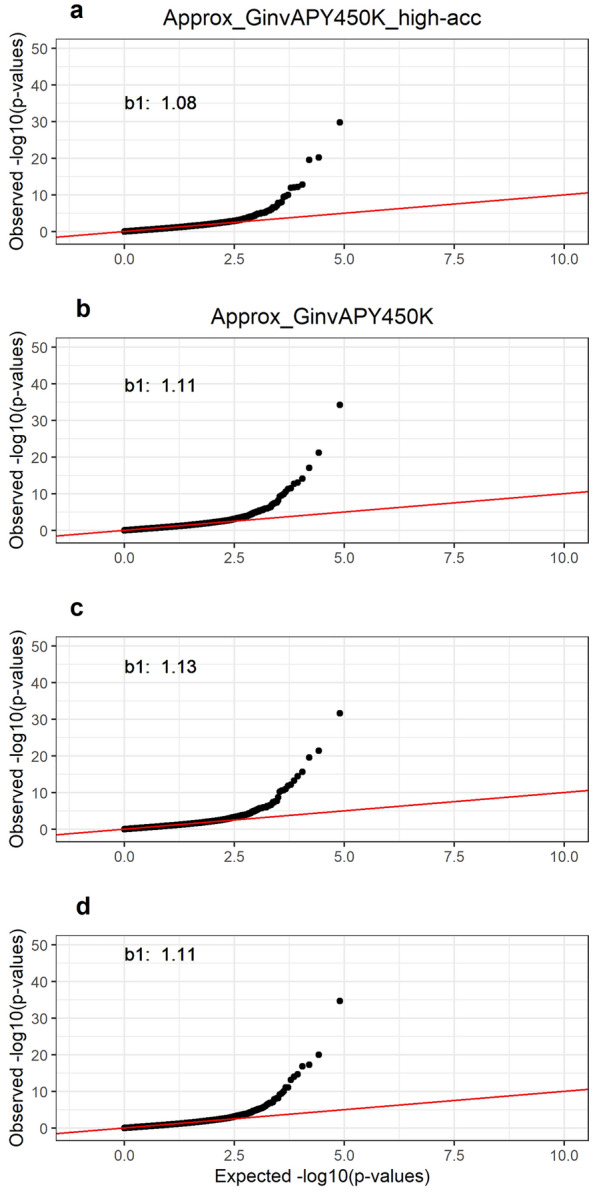
QQ plots for p-values obtained with Approx_GinvAPY450K and Approx_GinvAPY450K_high-acc. QQ plots for p-values obtained with Approx_GinvAPY450K in (**A**) replicate 1, (**B**) replicate 2, (**C**) replicate 3, and (**D**) with Approx_GinvAPY450K_high-acc. Approx_GinvAPY450K refers to the method where SNP p-values are obtained from a data set of 450K genotyped animals with ${\mathbf{G}}_{\mathbf{A}\mathbf{P}\mathbf{Y}}^\mathbf{-1}$ and ${\mathbf{C}}^{{{\mathbf{u}}_{\mathbf{2}_{\mathbf{C}}}}{\mathbf{u}}_{{\mathbf{2}_{\mathbf{C}}}_{{\varvec{a}}{\varvec{p}}{\varvec{p}}{\varvec{r}}{\varvec{o}}{\varvec{x}}}}}$ and where the APY core set if chosen at random; Approx_GinvAPY450K_high-acc refers to the Approx_GinvAPY450K when the APY core is composed of animals with the highest EBV accuracy in the population

Manhattan and QQ plots for p-values obtained with Approx_GinvAPY450K and Approx_GinvAPY450K_high-acc are displayed in Figs. 3 and 4, respectively. Across all replicates and scenarios, the two significant peaks on chromosomes 7 and 20 observed in the first set of analyses with Exact_Ginv (benchmark) were also identified with Approx_GinvAPY450K and Approx_GinvAPY450K_high-acc (Fig. 3). For the peak on chromosome 7, the same top three SNPs were identified with Approx_GinvAPY450K and Approx_GinvAPY450K_high-acc, which were consistent with benchmark results with the reduced dataset (i.e., Exact_Ginv). For the peak on chromosome 20, the first two top SNPs were consistent across Approx_GinvAPY450K and Approx_GinvAPY450K_high-acc while the third top SNP on chromosome 20 varied slightly across neighboring SNPs within a 0.13Mb window (Fig. 3). Results from QQ plots were also very similar between Approx_GinvAPY450K and Approx_GinvAPY450K_high-acc (Fig. 4). The slope of the QQ plot regression was 1.08 for Approx_GinvAPY450K_high-acc and 1.12 ± 0.01 across Approx_GinvAPY450K replicates, thus suggesting small influence of core composition on the deviation of p-values from the null hypothesis. Despite the consistency of results with different core composition shown in this study, previous experience with a single breed population showed that, especially with datasets with a clear unbalance of phenotypic and genotypic information (i.e., phenotypic dataset with several generations of recording combined with only recent generations contributing with genotypes), the selection of APY core based on a random selection always resulted in the best solutions in comparison to benchmark results with the exact inversion of the LHS [38]. However, a more informed choice of core animals [3, 39] may be used without considerable changes of p-values for the significant peaks in well-structured, single-breed populations. Note that the optimum core composition strategy can change in multi-breed populations, especially when breed contributions are highly unbalanced [21].

Enlarging the genotype set also uncovered two new peaks on chromosomes 6 and 14 that were not observed with the reduced dataset (Fig. 3). The new peaks had clear linkage disequilibrium trails, illustrating an increase in ssGWAS resolution as more genotyped animals are included in the analyses. As previously shown, especially for populations with a small effective population size (Ne) and more polygenic traits, increasing the genotype set reduces the estimation error and the shrinkage of SNP effects, which increases the power of discovering significant variants [34, 40, 41]. The benefit of an increase in the genotype set size can also be observed when comparing Approx_GinvAPY with Approx_GinvAPY450K. In general, for significant SNPs identified on chromosomes 7 and 20, the magnitude of p-values on the logarithmic scale obtained with Approx_GinvAPY450K increased by 50% relative to results from Approx_GinvAPY.

In the first set of analyses, when the same amount of data was used, an increase in noise was observed with Approx_GinvAPY compared to Exact_GinvAPY (Fig. 1, Additional file 1: Figures S1 and S2). However, when more genotyped animals were included with Approx_GinvAPY450K, significant SNPs without a clear linkage disequilibrium pattern were no longer observed in all replicates (Fig. 3). This suggests that the benefit of increasing the genotype set overcomes the noise associated with an approximation that relies on APY and ${\mathbf{C}}^{{{\mathbf{u}}_{\mathbf{2}_{\mathbf{C}}}}{\mathbf{u}}_{{\mathbf{2}_{\mathbf{C}}}_{{\varvec{a}}{\varvec{p}}{\varvec{p}}{\varvec{r}}{\varvec{o}}{\varvec{x}}}}}$ and mitigates potential false positive associations. While evaluating two simulated populations with the same Ne, Misztal et al. [34] observed that increasing the number of individuals contributing with genotypes and phenotypes by three times increased the correct identification of significant SNPs. Similarly, Jang et al. [40] showed that for highly polygenic traits (2000 QTN) with an Ne of 20 and a moderate heritability of 0.30, no QTN was accurately identified until a complete genotype set, composed of 30K genotyped animals, was included in the analyses. For livestock populations with even smaller Ne and traits of lower heritability, such as reproduction and fitness traits, QTN identification may be even more challenging, especially when limitations exist on the amount of genomic information used in the estimation process.

Total wall-clock time for the calculation of p-values with Approx_GinvAPY450K was, on average, 24.47h, which was divided into building ${\mathbf{G}}_{\text{APY}}^\mathbf{-1}$ and saving components of ${\mathbf{A}}_{22}^\mathbf{-1}$ (6.6h), estimation of breeding values (6.67h), estimation of ${\mathbf{C}}^{{{\mathbf{u}}_{\mathbf{2}_{\mathbf{C}}}}{\mathbf{u}}_{{\mathbf{2}_{\mathbf{C}}}_{{\varvec{a}}{\varvec{p}}{\varvec{p}}{\varvec{r}}{\varvec{o}}{\varvec{x}}}}}$ (0.38h), and backsolving GEBV to SNP effects and approximation of $\text{var}\left({\widehat{\text{a}}}_{\text{i}}\right)$. The entire process required no more than 87.64GB of RSS memory (Table 4). In comparison with the same method using a reduced set of genotyped animals in the first set of analyses (i.e., Approx_GinvAPY), the increase in wall-clock time was linear with the increase in the number of genotypes included added, which was approximately nine times. However, the increase in RSS memory requirement was only five times.

**Table 4:** Elapsed wall-clock time and Resident Set Size (RSS) requirements for p-values computation with Approx_GinvAPY450K^a^

Software	Elapsed time, h:min	SD	Peak of memory, GB^b^	SD
PREGSF90	6:36	0:13	51.37	0.00
BLUPF90IOD3	6:40	0:31	43.82	0.00
ACCF90GS2	0:23	0.02	87.64	0.00
POSTGSF90	10:48	0.40	59.43	0.87
Total/Max	24:28		87.64	

^a^SNP p-values obtained from a data set of 450K genotyped animals with ${\mathbf{G}}_{\mathbf{A}\mathbf{P}\mathbf{Y}}^\mathbf{-1}$ and ${\mathbf{C}}^{{{\mathbf{u}}_{\mathbf{2}_{\mathbf{C}}}}{\mathbf{u}}_{{\mathbf{2}_{\mathbf{C}}}_{{\varvec{a}}{\varvec{p}}{\varvec{p}}{\varvec{r}}{\varvec{o}}{\varvec{x}}}}}$(Approx_GinvAPY)

^b^Resident Set Size (RSS) memory. Values are displayed as average and standard deviations between three replicates

The efficiency of the proposed approximation method (i.e., Approx_GinvAPY) is because $\text{var}\left({\widehat{\text{a}}}_{\text{i}}\right)$ computations rely only on the genotypes of core animals, meaning that the computational requirement of inverting $\mathbf{G}$ in Exact_Ginv and obtaining ${\mathbf{G}}_{\mathbf{A}\mathbf{P}\mathbf{Y}}^\mathbf{-1}$ in Exact_GinvAPY is reduced to inverting a small matrix of relationships between core animals (${\mathbf{G}}_{\mathbf{c}\mathbf{c}}$) [16].

The optimal dimension of ${\mathbf{G}}_{\mathbf{c}\mathbf{c}}$ is approximately a linear function of Ne of the population and should not be more than 15K for most livestock species or breeds [21, 22]. Moreover, with the Approx_GinvAPY method, no inversion of the LHS is required. Instead, ${\mathbf{C}}^{{{\mathbf{u}}_{\mathbf{2}_{\mathbf{C}}}}{\mathbf{u}}_{{\mathbf{2}_{\mathbf{C}}}_{{\varvec{a}}{\varvec{p}}{\varvec{p}}{\varvec{r}}{\varvec{o}}{\varvec{x}}}}}$ are obtained accurately with a lower computational cost by a block sparse inversion of ${\mathbf{G}}_{\mathbf{A}\mathbf{P}\mathbf{Y}}^\mathbf{-1}$ that had weights (effective record contributions; **D** in Eq. [9]) added to its diagonal [18]. Additionally, because Approx_GinvAPY does not require the direct inversion of the LHS, efficient solvers such as PCG can be used in combination with the block implementation of APY, efficiently exploiting the sparseness of ${\mathbf{G}}_{\mathbf{A}\mathbf{P}\mathbf{Y}}^\mathbf{-1}$[36, 37].

Even though this study focuses on combining APY and ${\mathbf{C}}^{{{\mathbf{u}}_{\mathbf{2}_{\mathbf{C}}}}{\mathbf{u}}_{{\mathbf{2}_{\mathbf{C}}}_{{\varvec{a}}{\varvec{p}}{\varvec{p}}{\varvec{r}}{\varvec{o}}{\varvec{x}}}}}$[18], any efficient method to approximate the GEBV prediction error covariance or SNP prediction error variances in large genotyped populations could be applied here. For a comparison of APY against other methods, we refer the reader to Bermann et al. [18] and Zaabza et al. [42].

Altogether, our results show that the current computational limitations for obtaining p-values for populations with many genotyped animals should no longer be an issue with the Approx_GinvAPY method. The possibility of computing SNP p-values for those large genotyped populations should increase the power of detection of true variants and prevent future findings of ssGWAS from solely relying on SNP effects and variance explained by SNPs [7, 11]. It is worth noting that the results presented herein are based on a single-trait model in a purebred population. However, as long as reliabilities from more complex models and on populations with more complex breeding structures are accurately estimated, we expect that p-values will also be accurately approximated.

## Conclusions

The same genomic regions on chromosomes 7 and 20 were identified with p-values obtained with ${\mathbf{G}}^\mathbf{-1}$, ${\mathbf{G}}_{\mathbf{A}\mathbf{P}\mathbf{Y}}^\mathbf{-1}$, and the approximation based on ${\mathbf{G}}_{\mathbf{A}\mathbf{P}\mathbf{Y}}^\mathbf{-1}$ with a reduced dataset, indicating the soundness of the proposed p-value computing method. Even though p-values were similar between computing methods, computational requirements for the new method were considerably reduced. When the approximation based on ${\mathbf{G}}_{\mathbf{A}\mathbf{P}\mathbf{Y}}^\mathbf{-1}$ was applied to a genotyped population with almost half a million genotyped animals, SNPs on chromosomes 7 and 20 had stronger signals, and two new regions on chromosomes 6 and 14 were uncovered, indicating an increase in ssGWAS detection power when more genotypes are included in the analyses. Obtaining p-values in ssGWAS for such a large genotyped population required 24h, which is expected to increase linearly with the addition of noncore genotyped individuals. With a combination of APY and an approximation of the variance of estimated SNP effects, ssGWAS with p-values becomes computationally feasible for large genotyped populations.

### Supplementary Information

**Additional file 1**: Figure S1. Manhattan plots for all p-value computing methods with a reduced data set in replicate 2. Single-step genome-wide association study for post-weaning weight with p-values obtained from a data set of 50K genotyped animals with (A) ${\mathbf{G}}^\mathbf{-1}$ and ${\mathbf{C}}^{{\mathbf{u}}_\mathbf{2}{\mathbf{u}}_\mathbf{2}}$ (Exact_Ginv), (B) ${\mathbf{G}}_{\mathbf{A}\mathbf{P}\mathbf{Y}}^\mathbf{-1}$ and ${\mathbf{C}}^{{{\mathbf{u}}_{\mathbf{2}_{\mathbf{C}}}}{\mathbf{u}}_{\mathbf{2}_{\mathbf{C}}}}$(Exact_GinvAPY), and (C) ${\mathbf{G}}_{\mathbf{A}\mathbf{P}\mathbf{Y}}^\mathbf{-1}$ and ${\mathbf{C}}^{{{\mathbf{u}}_{\mathbf{2}_{\mathbf{C}}}}{\mathbf{u}}_{{\mathbf{2}_{\mathbf{C}}}_{{\varvec{a}}{\varvec{p}}{\varvec{p}}{\varvec{r}}{\varvec{o}}{\varvec{x}}}}}$(Approx_GinvAPY) in replicate 2; SNPs highlighted in green represent the three most significant SNP in the two peaks found with Exact_Ginv (benchmark). Figure S2. Manhattan plots for all p-value computing methods with a reduced data set in replicate 3. Single-step genome-wide association study for post-weaning weight with p-values obtained from a data set of 50K genotyped animals with (A) ${\mathbf{G}}^\mathbf{-1}$ and ${\mathbf{C}}^{{\mathbf{u}}_\mathbf{2}{\mathbf{u}}_\mathbf{2}}$ (Exact_Ginv), (B) ${\mathbf{G}}_{\mathbf{A}\mathbf{P}\mathbf{Y}}^\mathbf{-1}$ and ${\mathbf{C}}^{{{\mathbf{u}}_{\mathbf{2}_{\mathbf{C}}}}{\mathbf{u}}_{\mathbf{2}_{\mathbf{C}}}}$(Exact_GinvAPY), and (C) ${\mathbf{G}}_{\mathbf{A}\mathbf{P}\mathbf{Y}}^\mathbf{-1}$ and ${\mathbf{C}}^{{{\mathbf{u}}_{\mathbf{2}_{\mathbf{C}}}}{\mathbf{u}}_{{\mathbf{2}_{\mathbf{C}}}_{{\varvec{a}}{\varvec{p}}{\varvec{p}}{\varvec{r}}{\varvec{o}}{\varvec{x}}}}}$(Approx_GinvAPY) in replicate 3; SNPs highlighted in green represent the three most significant SNP in the two peaks found with Exact_Ginv (benchmark). Figure S3. QQ plots for all p-value computing methods with a reduced data set in replicate 2. QQ plots for p-values obtained from a data set of 50K genotyped animals with (A) ${\mathbf{G}}^\mathbf{-1}$ and ${\mathbf{C}}^{{\mathbf{u}}_\mathbf{2}{\mathbf{u}}_\mathbf{2}}$ (Exact_Ginv), (B) ${\mathbf{G}}_{\mathbf{A}\mathbf{P}\mathbf{Y}}^\mathbf{-1}$ and ${\mathbf{C}}^{{{\mathbf{u}}_{\mathbf{2}_{\mathbf{C}}}}{\mathbf{u}}_{\mathbf{2}_{\mathbf{C}}}}$(Exact_GinvAPY), and (C) ${\mathbf{G}}_{\mathbf{A}\mathbf{P}\mathbf{Y}}^\mathbf{-1}$ and ${\mathbf{C}}^{{{\mathbf{u}}_{\mathbf{2}_{\mathbf{C}}}}{\mathbf{u}}_{{\mathbf{2}_{\mathbf{C}}}_{{\varvec{a}}{\varvec{p}}{\varvec{p}}{\varvec{r}}{\varvec{o}}{\varvec{x}}}}}$(Approx_GinvAPY) in replicate 2. Figure S4. QQ plots for all p-value computing methods with a reduced data set in replicate 3. QQ plots for p-values obtained from a data set of 50K genotyped animals with (A) ${\mathbf{G}}^\mathbf{-1}$ and ${\mathbf{C}}^{{\mathbf{u}}_\mathbf{2}{\mathbf{u}}_\mathbf{2}}$ (Exact_Ginv), (B) ${\mathbf{G}}_{\mathbf{A}\mathbf{P}\mathbf{Y}}^\mathbf{-1}$ and ${\mathbf{C}}^{{{\mathbf{u}}_{\mathbf{2}_{\mathbf{C}}}}{\mathbf{u}}_{\mathbf{2}_{\mathbf{C}}}}$(Exact_GinvAPY), and (C) ${\mathbf{G}}_{\mathbf{A}\mathbf{P}\mathbf{Y}}^\mathbf{-1}$ and ${\mathbf{C}}^{{{\mathbf{u}}_{\mathbf{2}_{\mathbf{C}}}}{\mathbf{u}}_{{\mathbf{2}_{\mathbf{C}}}_{{\varvec{a}}{\varvec{p}}{\varvec{p}}{\varvec{r}}{\varvec{o}}{\varvec{x}}}}}$(Approx_GinvAPY) in replicate 3.

## Data Availability

The data supporting the findings of this study were provided from the American Angus Association (St. Joseph, MO) but restrictions apply to the availability of these data, which were used under license for the current study, and thus are not publicly available. The methods described here when using ${\mathbf{G}}^\mathbf{-1}$ and ${\mathbf{G}}_{\mathbf{A}\mathbf{P}\mathbf{Y}}^\mathbf{-1}$ are included in BLUPF90 + and POSTGSf90, available at http://nce.ads.uga.edu/software/. The methods described here when using the approximation based on ${\mathbf{G}}_{\mathbf{A}\mathbf{P}\mathbf{Y}}^\mathbf{-1}$ are included in BLUP90IOD3 and ACCF90GS, which are only available under research agreement with the Animal Breeding and Genetics group at UGA (http://nce.ads.uga.edu/).
