# Supplementary figures and images for: Marker effect p-values for single-step GWAS with the algorithm for proven and young in large genotyped populations
Source: Genet Sel Evol. 2024 Aug 22;56:59. doi: 10.1186/s12711-024-00925-3 (PMC11340074; doi:10.1186/s12711-024-00925-3)

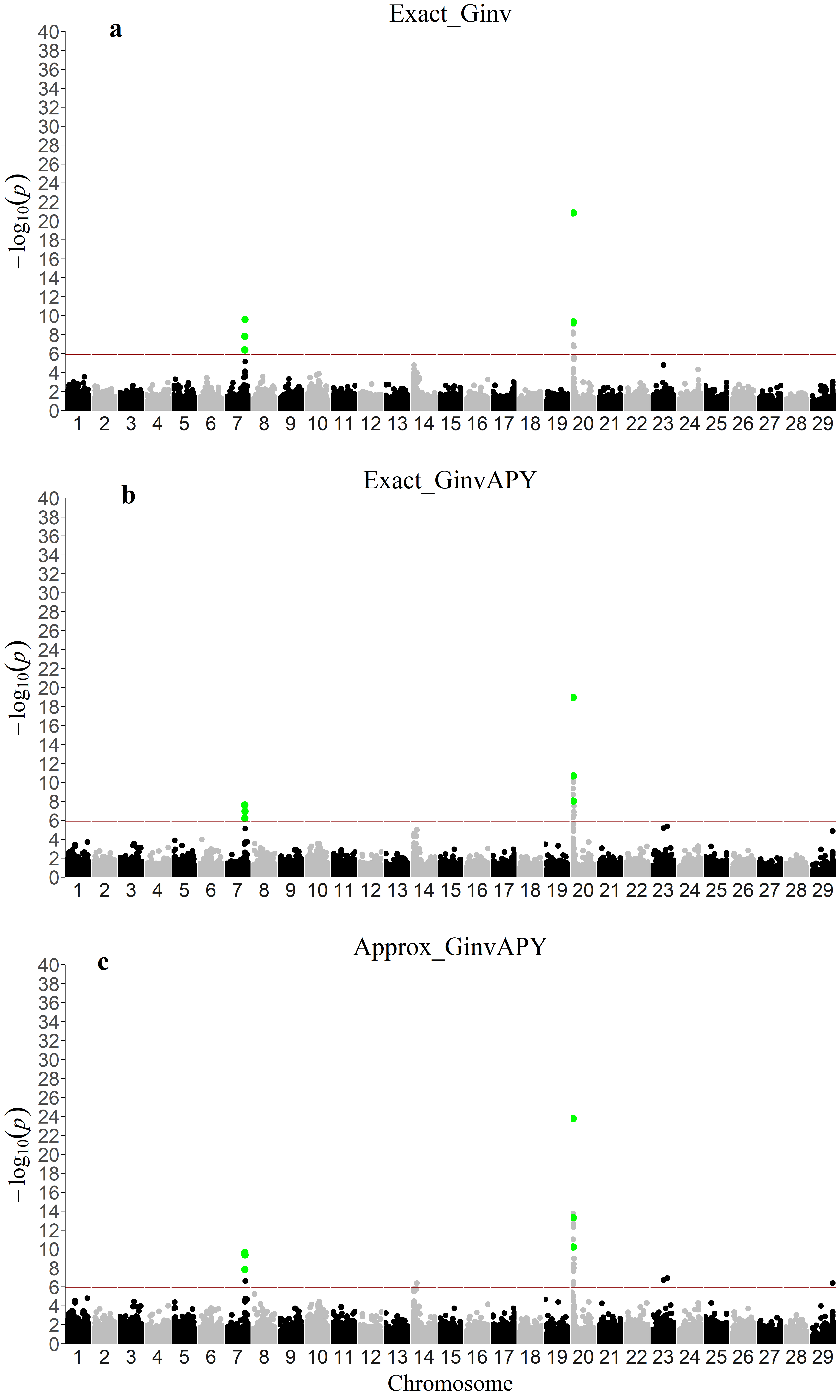
 **Figure S1**

**Figure S2**


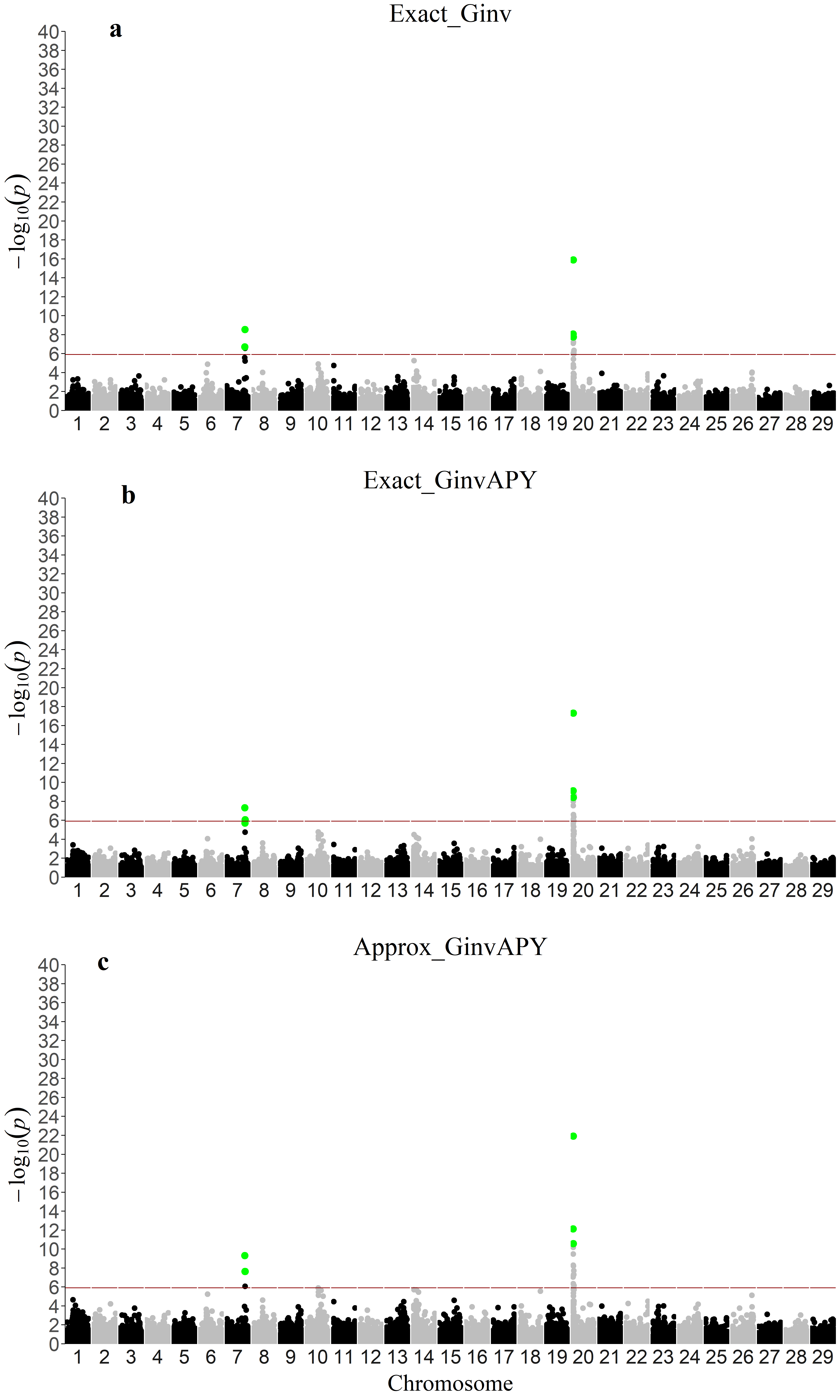


**Figure S3**


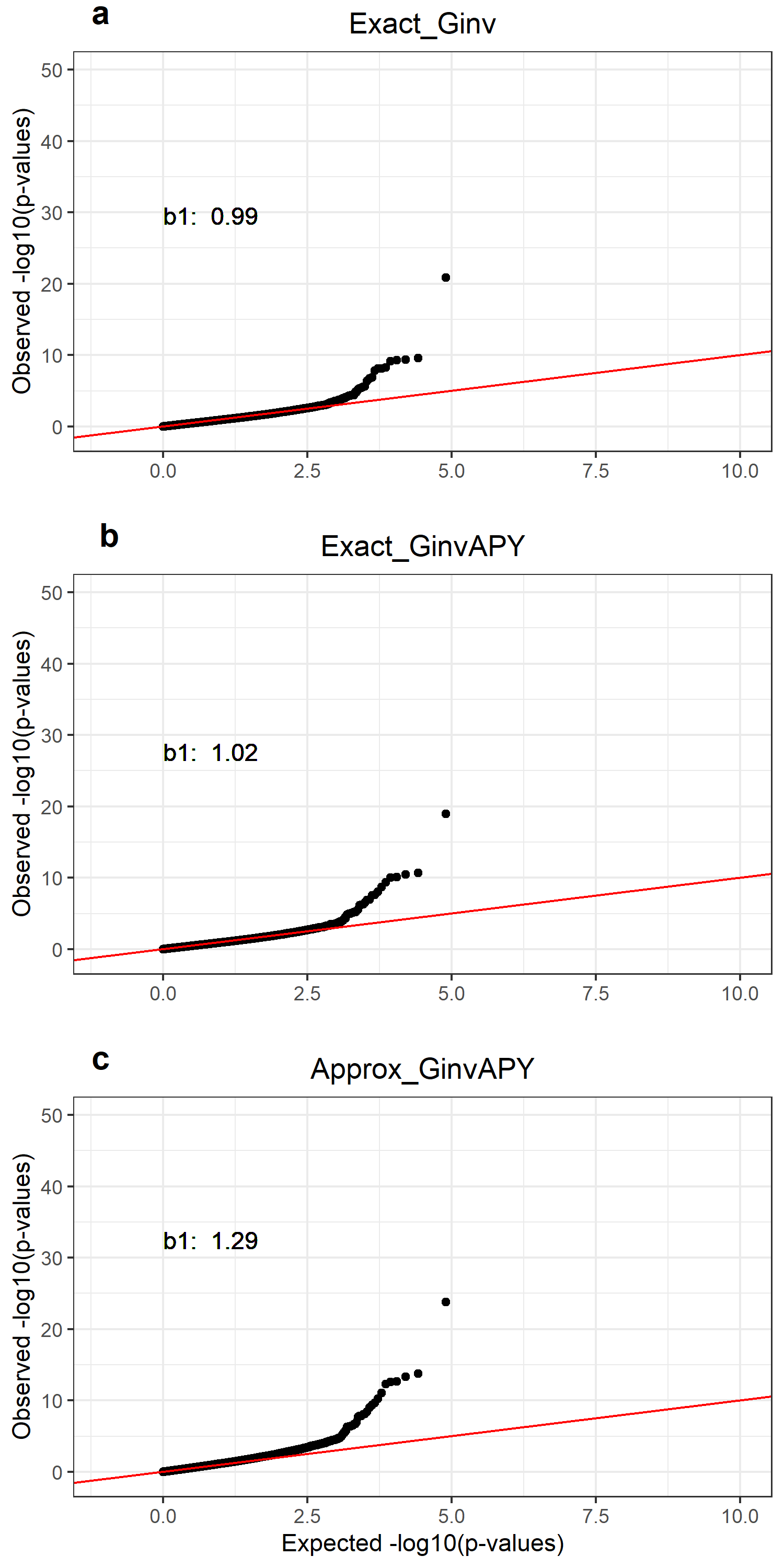


**Figure S4**


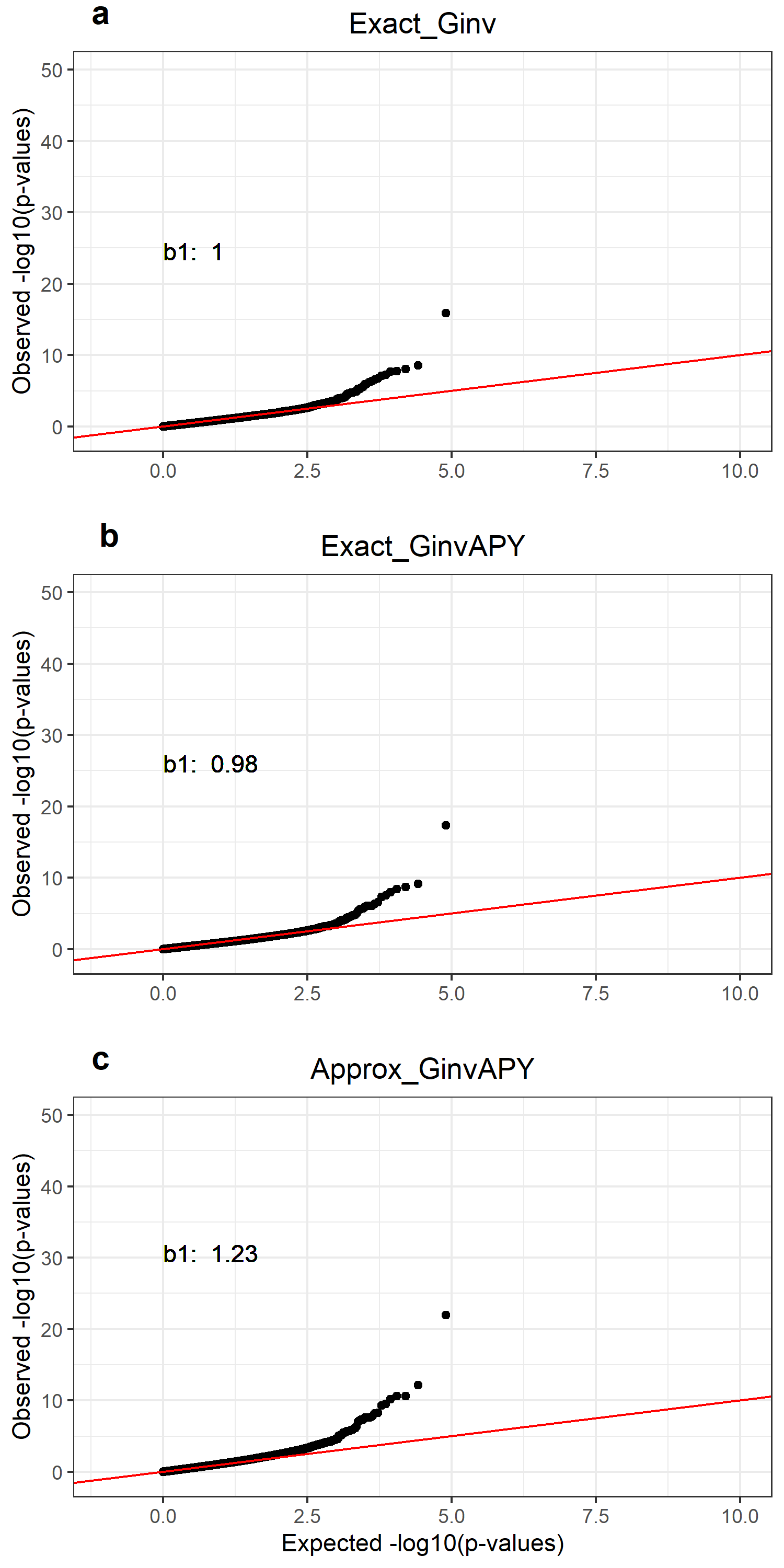

Supplement: Supplementary file 1 — Additional file 1: Figure S1. Manhattan plots for all p-value computing methods with a reduced data set in replicate 2. Single-step genome-wide association study for post-weaning weight with p-values obtained from a data set of 50K genotyped animals with (A) ${\mathbf{G}}^\mathbf{-1}$G-1 and ${\mathbf{C}}^{{\mathbf{u}}_\mathbf{2}{\mathbf{u}}_\mathbf{2}}$Cu2u2 (Exact_Ginv), (B) ${\mathbf{G}}_{\mathbf{A}\mathbf{P}\mathbf{Y}}^\mathbf{-1}$GAPY-1 and ${\mathbf{C}}^{{{\mathbf{u}}_{\mathbf{2}_{\mathbf{C}}}}{\mathbf{u}}_{\mathbf{2}_{\mathbf{C}}}}$Cu2Cu2C(Exact_GinvAPY), and (C) ${\mathbf{G}}_{\mathbf{A}\mathbf{P}\mathbf{Y}}^\mathbf{-1}$GAPY-1 and [file 12711_2024_925_MOESM1_ESM.docx]
